# Effect of Supplementation with Olive Leaf Extract Enriched with Oleuropein on the Metabolome and Redox Status of Athletes’ Blood and Urine—A Metabolomic Approach

**DOI:** 10.3390/metabo12020195

**Published:** 2022-02-20

**Authors:** Nikolaos Lemonakis, Vassilis Mougios, Maria Halabalaki, Ioanna Dagla, Anthony Tsarbopoulos, Alexios-Leandros Skaltsounis, Evagelos Gikas

**Affiliations:** 1 Department of Pharmacognosy and Natural Products Chemistry, Faculty of Pharmacy, National and Kapodistrian University of Athens, Panepistimioupolis Zografou, 157 71 Athens, Greece; nlemonakis@pharm.uoa.gr (N.L.); mariahal@pharm.uoa.gr (M.H.); skaltsounis@pharm.uoa.gr (A.-L.S.); 2 Laboratory of Evaluation of Human Biological Performance, School of Physical Education and Sport Science, Aristotle University of Thessaloniki, 541 24 Thessaloniki, Greece; mougios@phed.auth.gr; 3 The Goulandris Natural History Museum, Bioanalytical Laboratory, GAIA Research Center, 145 62 Kifissia, Greece; idagla@pharm.uoa.gr (I.D.); atsarbop@med.uoa.gr (A.T.); 4 Department of Pharmacology, Medical School, National and Kapodistrian University of Athens, 115 27 Athens, Greece; 5 Laboratory of Analytical Chemistry, Faculty of Chemistry, National and Kapodistrian University of Athens, Panepistimioupolis Zografou, 157 71 Athens, Greece

**Keywords:** oleuropein, metabolomics, high-resolution mass spectrometry, multilevel-sPLSDA

## Abstract

Oleuropein (OE) is a secoiridoid glycoside occurring mostly in the Oleaceae family and presenting several pharmacological properties, including hypolipidemic and antioxidant properties. Based on these, several dietary supplements containing olive leaf extracts enriched with OE are commercially available in many countries. The current study aimed to examine the effect of supplementation with such an extract on the serum and urine metabolome of young healthy male athletes. For this purpose, applying a randomized, balanced, double-blind study, nine young, healthy males (physical education students) received either a commercially prepared extract or placebo for one week, followed by a two-week washout period; then, they were subsequently dosed with the alternate scheme (crossover design). Urine and serum samples were analyzed using UHPLC-HRMS, followed by evaluation with several multivariate methods of data analysis. The data were interpreted using a multilevel metabolomic approach (multilevel-sPLSDA) as it was found to be the most efficient approach for the study design. Metabolic pathway analysis of the most affected metabolites revealed that tryptophan and acylcarnitine’s biochemistries were most influenced. Furthermore, several metabolites connected to indole metabolism were detected, which may indicate enhanced serotonin turnover. Phenylethylamine and related metabolites, as well as estrone, were connected to enhanced performance. In addition, possible changes to the lipidemic profile and the blood and urine redox statuses were investigated.

## 1. Introduction

Oleuropein (OE) is a nontoxic natural secoiridoid glycoside, occurring mainly in the *Olea* genus of the *Oleaceae* family. It is present in large amounts in olive leaves and in smaller quantities in olive oil and table fruits. It is the most well-studied compound in olive cultivars [1,2] and is considered one of the most health-promoting compounds of the Mediterranean diet. It has been proven to exhibit protective activity against an array of common chronic pathological conditions [3,4], e.g., with antioxidant [5], antimicrobial [6], cardioprotective [7,8], anti-ischemic [9], antiatherogenic [10], anti-inflammatory [11,12], antidiabetic [13] and anti-atherosclerotic [14,15] properties, among others. Especially concerning its antioxidant activity, several in vitro studies have demonstrated that OE possesses high antioxidant activity, comparable to a hydrosoluble analog of tocopherol [9].

Recently, the European Food Safety Authority (EFSA) endorsed the health claim that ‘‘the consumption of olive oil polyphenols contributes to the protection of blood lipids to oxidative damage’’ [16]. Thus, several formulations of olive leaf extracts enriched in OE are being made commercially available in many countries as food supplements or herbal medicinal products [17].

Oxidative stress can be defined as an increase of the intracellular steady-state concentration of oxidants over physiological values [18,19,20,21]. As physical exercise may enhance the oxidative stress of an organism due to elevated catabolism that may produce large amounts of oxidized substances, athletes can be considered an ideal study group. Following feedback regulation, athletes under a regular training program may exhibit a significant increase in antioxidant status compared to healthy sedentary people [22], due to the adaptative response to controlled physical activity. Nevertheless, during overload training, the organism’s adaptative response cannot efficiently control free radical production, which could prove detrimental to the body [23]. At the start of an exercise program, muscle damage in athletes frequently occurs [24], often attributed to an intensity-dependent increase in oxidative stress produced by mitochondrial processes [25]. Small increases in exercise induce increased production of reactive oxygen species (ROS), an important process for stimulating cellular growth and maximizing muscular force production, whereas excessive accumulation leads to a pro-oxidant environment, which can damage DNA. Rapid recovery from the prior exercise is important for both beginners performing regular exercise to improve their health and athletes preparing for competition. Thus, antioxidant supplementation to attenuate exercise-induced muscle injury, inflammation and pain may facilitate success in both health promotion and sport competition.

The World Health Organization (WHO) has introduced the Global Recommendations on Physical Activity for Health [26], which encourage people of all ages to begin moderately and gradually progressing to higher levels of physical activity. Thus, antioxidants may be administrated before any exercise, especially before high-level training, and result in the annihilation of the free radical production generated by the exercise that potentially overwhelms the defensive mechanisms, causing oxidative stress [27,28]. Different types of supplementation such as vitamin E [29], vitamin C [30] or polyphenols show that it is possible to enhance the adapted antioxidant status. However, studies that evaluated the impact of the antioxidant effect on the performance or endurance of athletes revealed various results that differed largely, depending on the type of supplementation, subjects and protocol [31].

Only a few studies have been performed in human subjects concerning the antioxidant effects of OE. In these, olive (*Olea europaea* L.) leaf phenols were found to improve insulin sensitivity in middle-aged overweight men in a randomized, placebo-controlled, crossover trial [32], and a possible association between the consumption of rich-phenol extra virgin olive oil and an enhancement of the endogen antioxidant system in healthy elderly people was postulated [33]. Furthermore, it was demonstrated that young and middle-aged men and women who consumed a moderate amount of extra-virgin olive oil (EVOO) regularly increased their antioxidant status [34,35]. The aforementioned positive effects of EVOO have been attributed to its high phenolic content and especially its high level of secoiridoid derivatives [36,37]. In this context, a randomized, double-blinded, placebo-controlled, crossover trial encompassing supplementation with olive leaf extract enriched in OE was designed.

Nowadays, crossover designs are used in combination with ‘omics’, resulting in paired multivariate datasets [38,39,40]. Metabolomic time-series experiments with different subjects use the same paired data structure [41,42], and the analysis of such datasets can possibly be improved when exploiting the design underlying the study, a fact that is not always considered in such analyses.

One of the major challenges in crossover supplementation/nutrition metabolomic studies is the detection and identification of metabolites in different biofluids, such as serum and urine, which can be linked to possible changes in the human metabolome after the supplementation period [43,44,45]. In such studies (as in the current one), the metabolic responses observed are usually small since the volunteers are in metabolic homeostasis, i.e., they are generally in a healthy state [46]. Thus, since the biological variation between individuals is usually much larger than the effects of administration, small and subtle treatment effects, i.e., dietary responses can be easily overlooked. This is notable when the effect is smaller than the intrinsic variation between the subjects, whereas on other occasions, the response may differ significantly between subjects. This implies that the average treatment effect may not be the most prevailing in studies where subsets of subjects respond differently to a dietary intervention. Therefore, a frequently used solution, followed in clinical or nutritional intervention studies, is a crossover design, with subjects acting as their own controls and the total variance broken down into constituting ones (e.g., between-subject variation, within-subject values’ variation, etc.), to uncover minor treatment effects. The traditional multivariate data analysis methods (PCA, PLS, PLS-DA, etc.) do not consider the decomposition of the total variance; therefore, subtle treatment effects experienced by subjects are often largely overwhelmed by the strong biological variation between subjects [47]. The combination of multilevel data analysis, i.e., decomposing the total variation and PLS-DA, is introduced as a new MVA approach to investigate treatment effects in crossover-designed experiments [40].

The current study aimed to evaluate the effects of supplementation with an OE-enriched olive leaf extract pharmaceutical product on the serum and urine metabolomes of young, healthy male athletes, employing a randomized, double-blind, crossover design. It was assumed that supplementation with OE would improve the antioxidant status of the athletes and would have a positive antioxidant impact on their recovery after a training period, preventing the occurrence of DNA damage.

## 2. Results and Discussion

### 2.1. Biochemical Analyses

A battery of classical biochemical analyses found that olive leaf extract systematically changes the levels of triglycerides in plasma but only sporadically changes the levels of total cholesterol, HDL cholesterol and LDL cholesterol.

### 2.2. UHPLC-HRMS Analysis—Data Preprocessing

#### 2.2.1. Peak Picking Procedure

Two algorithms, “baseline cut-off” and “wavelets”, were evaluated for facilitating metabolite detection by MZmine. For this purpose, the xMSanalyzer R package was used to compare the results of the two procedures. A Venn diagram was constructed, showing both common and unique features (Figure SA1). From a total number of 1492 features discovered, 887 (60%) were common, 203 (13%) were unique for the “baseline cut-off” and 402 (27%) for the “wavelets” algorithm. The common features were assembled in a peak list and submitted for further evaluation.

#### 2.2.2. Metaboanalyst Process

Missing values were imputed by employing PPCA [48], whereas the option of “normalization by a reference sample”, specifically by averaging all the samples (using a pseudo-reference sample in the control group), was chosen for the normalization of the data.

### 2.3. MVA Analysis

**Figure 1:**
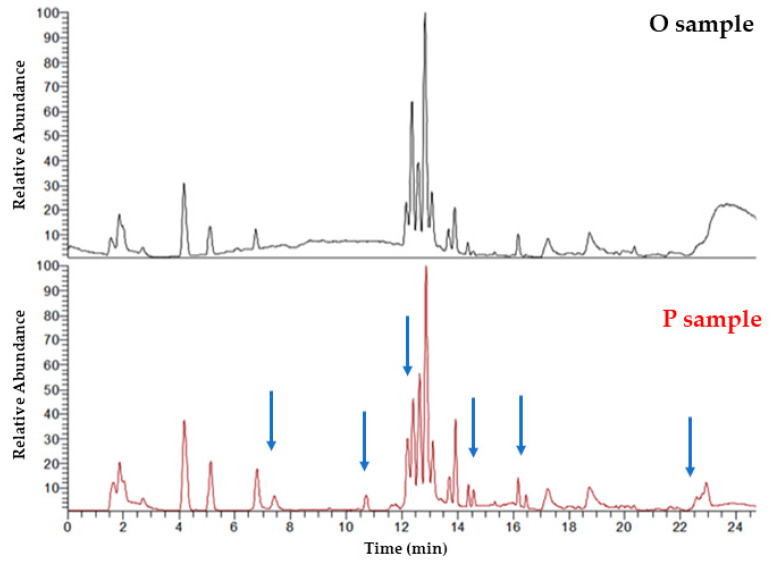
Representative BPI UHPLC-(+)ESI-HRMS serum chromatograms from individual B in the first week of administration (top) in juxtaposition with the second week of administration (placebo administration) (below). Differences can be observed (blue arrows) by visual inspection and offer complementary information regarding the metabolite profiles.

Representative base peak intensity (BPI) UHPLC-(+)ESI-HRMS serum chromatograms from individual B in the first week of administration in juxtaposition with the second week of administration (placebo administration) are shown in Figure 1. Differences in metabolic profiles due to the administration of oleuropein (O) vs. placebo (P) can be observed by visual inspection.

MVA was employed using various statistical models, such as PCA, siPCA, sPCA, PLS-DA, OPLS-DA, sPLS-DA and ML-sPLS-DA (k-PCA, SVM Random Forests, hierarchical/KNN clustering), the impact of the group/class they belong to (e.g., O vs. P or O vs. baseline (B)) the days of administration (e.g., all the days of administration, first day vs. last day, two last days) and the individuality (e.g., all the subjects simultaneously or one individual at a time).

Due to the large biological variation between the individuals, nine PCA models were created for each subject, i.e., between the two weeks of crossover administration (P vs. O) (Table SB1). The P group was clustered separately from O for each subject (Figure SA2). This was repeated for all the urine and serum datasets. Nevertheless, the large biological variation superseded the respective variation caused by the administration effect and hindered the PCA clustering capacity to create an overall model.

Other PCA variants, i.e., sPCA and siPCA, were employed to take advantage of sparse models and k-PCA with numerous kernels (Gaussian, polynomial, linear, hyperbolic tangent, Laplacian Bessel, ANOVA RBF and spline), but all efforts were unsuccessful at obtaining an overall model.

#### 2.3.1. PLS-DA and OPLS-DA Models

PLS-DA and OPLS-DA models were generated between P and O dosed subjects, between the subjects before (B) and after administration (P or O samples) and for B vs. O or P vs. O on selected days of administration (first vs. last day of administration) (Figures SA3–SA5). These models did not lead to any significant clustering (urine and serum in both positive and negative ion modes).

#### 2.3.2. PLS-DA and OPLS-DA Models

As the variation between the subjects usually outweighs the administration effects [39], which are typically small, the classical MVA approaches (PCA, PLS-DA, OPLS-DA, etc.) did not lead to any results. Thus, an ML-PLS-DA was performed for the serum and urine. In crossover studies, two main variation types could be observed, the biological (between the subjects) and the variation induced by the administration of OE (within the subjects). Other types of variation could also be recognized in the current study: the variation caused by the week of administration (before or after the washout period), i.e., the week-based effect; the variation caused by the day of administration (cumulative effect), i.e., the day-based effect and the analytical variation, i.e., the analytical error. The total variation results from the outcome of the aforementioned individual variations, as follows:

Var = Σ(Var_within_ + Var_between_ + Var_week_ + Var_day_ + Var_analytical error_) (1)

The ML-sPLS-DA approach was employed to break down the total variance into singular variations, enabling the identification of the individual effects (e.g., the between-subject variation, the week-based effect, etc.) on the response.

Four effects—the between (Var_between_) and within (Var_within_)-subject variations and the week-based (Var_week_) and day-based (Var_day_) effects—were recognized as discriminating variables, besides the analytical error. Thus, the discriminating variables used were based on (1) the between-subject variation (from A to I for the nine individual subjects), (2) the within-subject variation (P or O for placebo or oleuropein administration, respectively), (3) the week-based effect (one to two for the first or second week of administration) and (4) the day-based effect (from one to five for the different days of P or O administration during the two weeks of the crossover design).

**Figure 2:**
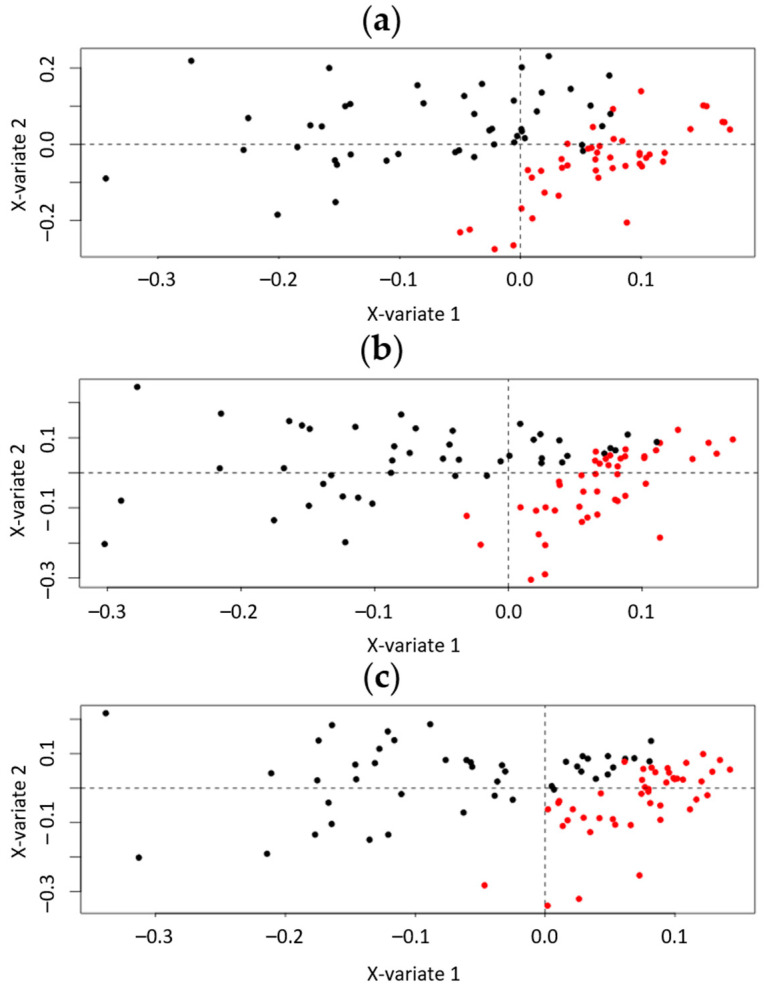
One-level ML-sPLS-DA analysis of the P and O urine samples from all the days of administration, obtained by UHPLC-(+)ESI-HRMS. The multilevel PLS-DA scores plot (X-variate 1, X-variate 2) the administration effect of Var_within_ according to the (**a**) biological variation (Var_between_) (**b**) week-based effect (Var_week_) and (**c**) day-based effect (Var_day_). Red circles represent the P samples. Black circles represent the O samples. Clear clustering mainly due to the first component, between the P and O samples, is shown.

Initially, a one-level ML-sPLS-DA was applied to the datasets, keeping one variation as the discriminating variable (i.e., the within-subject variation, Var_within_) and subtracting another one simultaneously (i.e., the between-subject variation, Var_between_), to find out which variation had the greatest impact to the datasets. The clustering, examined by visual inspection, and the tune parameter values resulting from the tuning procedure, were used as the selection criteria to evaluate the importance of each variance. As an example, the one-level ML-sPLS-DA scores plot of P and O urine samples from all the days of administration, with the biological variation (Var_between_) to be subtracted obtained by UHPLC-(+)ESI-HRMS, is depicted in Figure 2a. The cross-validation tuning criterion afforded each sPLS-DA dimension a classification error of (0.12, 0.18, 0.21) for variable selection sizes of 50, 60 and 70 metabolites on each dimension. It is clearly shown that, by subtracting the biological variation of individuals from the total variation, the administration effect is evident, and the P (red) and O (black) samples can be adequately separated according to the first component (x-variate 1). Thus, when subtracting the correct variation that obscures the classification according to the selected variable (e.g., the administration of OE in the current case), a single component could be sufficient to assess the different treatment effects.

Subsequently, when leaving the within-subject variation Var_within_ (P or O administration) as the discriminate variable, as we did before, but subtracting the variation caused by the week of administration Var_week_ (week-based effect), it could be shown that the P (red) and O (black) samples clustered separately, denoting that the week-based effect also played a critical role in the classification. That could be possibly attributed to environmental changes during the weeks of the administration (e.g., the weather conditions), to differences during the sample handling, to storage instability or even to the washout period between the two weeks of administration. The cross-validation tuning criterion afforded an acceptable classification error for each sPLS-DA dimension (<0.22), for variable selection sizes of 50, 60 and 70 metabolites, respectively (Figure 2b).

Furthermore, to investigate the day-based effect on the within-subject variation, the variation caused by the day of the administration Var_day_ was subtracted (day-based effect), leaving, as before, the within-subject variation Var_within_ (different administration P or O) as the discriminate variable. Interestingly, the P (red) and O (black) samples clustered separately (Figure 2c) (cross-validation tuning classification error < 0.22).

**Figure 3:**
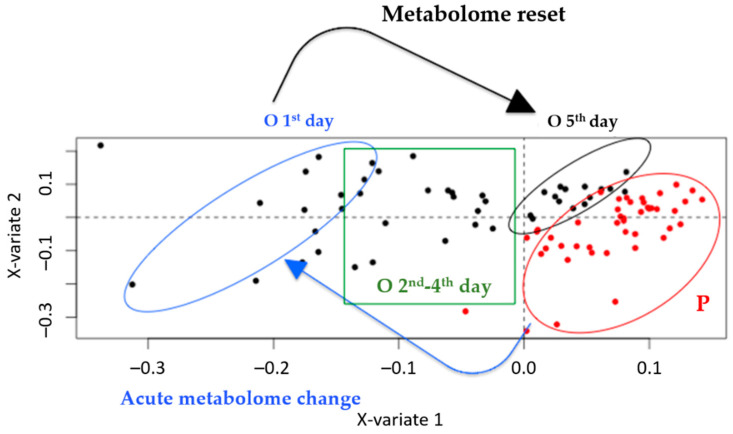
Annotated version of Figure 2c showing one-level ML-PLS-DA analysis of the P and O urine samples from all the days of administration, obtained by UHPLC-(+)ESI-HRMS. Red circles represent the P samples. Black circle represents the O samples from the fifth day of administration. Blue circle represents the O samples from the first day of administration. Green box represents O samples from the second to fourth days of administration. An acute metabolome change between P and O on the first day is evident (blue arrow), whereas a gradual metabolome reset can be observed (black arrow).

To explore the model in more detail, the administration days were annotated on the scores plot, emphasizing the two extremes of the administration i.e., the first vs. the last day (fifth) (Figure 3). It was evident that the samples from the fifth day were located closer to the P samples compared to the ones from the initial administration.

**Figure 4:**
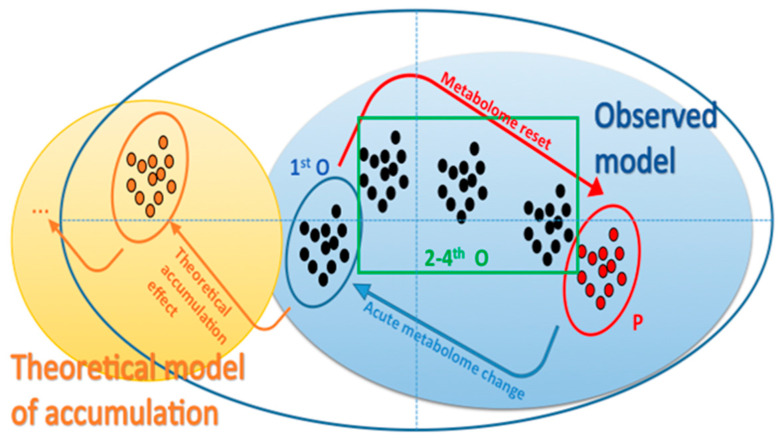
Theoretical model of accumulation vs. that observed in the one-level ML-PLS-DA approach.

The expected theoretical model predicted that repeated administration should produce a metabolic profile that would continuously differentiate after each administration from that of the control group (an accumulation effect), and ultimately, stabilize, rather than converging to that of the control group as in the current case. In contrast, the observed phenomenon was consistent with a reset of the metabolome to its initial state (Figure 4).

Overall, the week-based and day-based effects outweighed the effect caused by the administration, and hence, the subtraction of their variation would highlight the administration effect alone. A two-level ML-sPLS-DA approach was applied to the same datasets, keeping two variations as the discriminate variables and subtracting one. In other words, the algorithm used two discriminant values (e.g., week-based effect and administration effect) and it subtracted a third effect (e.g., biological variation effect) in the same calculation. Thus, a two-level ML-sPLS-DA analysis was performed with the same datasets, subtracting the between-subject variation Var_between_ from the total variance and keeping the combination of the within-subject variation and the day-based effect (Var_within_ + Var_day_) as the discriminant variables. As an example, the two-level ML-sPLS-DA 3D score plot of P and O urine samples from all the administration days (UHPLC-(+)ESI-HRMS) is depicted in Figure SA6. As is demonstrated in Figure SA6, the P and O samples are separated into two clusters, exhibiting a strong effect of the administration on the metabolism of the subjects. The validation-tuning criterion afforded acceptable maximum correlations of 0.85, 0.83 and 0.85 for each sPLS-DA dimension using variable selection sizes of 50, 60 and 70 metabolites, respectively, for each dimension. It seemed that repetitive dosing did not create an accumulation effect as no trend regarding the administration days was evident. In that case, one would expect that the metabolome should migrate further away from the placebo samples in a day-dependent fashion (the second day further than the first one, the third further than the second, etc.). On the contrary, the samples of the fourth and the fifth day seemed to retract to the placebo samples, as shown in Figure 3. A possible explanation could be that some kind of resistance due to enzyme induction was created (as O could be considered as a xenobiotic). It should also be noted that a three-day period is possibly a short time for observing a CYP induction or a microbiome-related effect concerning the degradation of OE. Thus, a different administration scheme should be considered, either encompassing lower dosing or diminishing the frequency of the administration. Nevertheless, this could be considered as a paradigm of using metabolomics in the pharmacology field.

**Figure 5:**
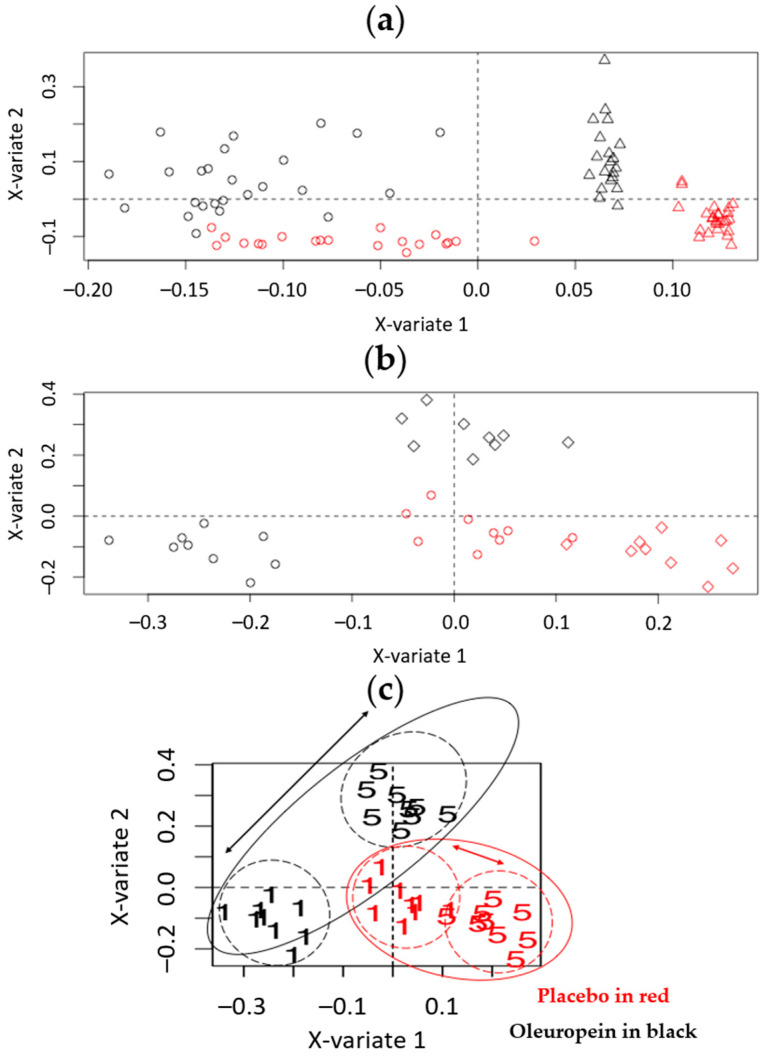
Two-level ML-PLS-DA analysis of the P and O urine samples analyzed by UHPLC-(+)ESI-HRMS (**a**) from all the days of administration (combination of the within-subject variation and the week-based effect (Var_within_ + Var_week_) as the discriminant values, with the day-based effect (Var_day_) subtracted; (**b**) from the two extremes of the administration days, i.e., the first vs. the last (fifth) (the combination of the within-subject variation and the day-based effect (Var_within_ + Var_day_) with the biological variation (Var_between_) subtracted). Then, (**c**) is an annotated version of panel (**b**). In all cases, black represents the O samples and red the P samples. In panel (**a**), circles represent the samples from the first week of administration and triangles represent the samples from the second week of administration. A clear clustering between the first and the second week due to the first component, and between the P and O samples due to the second component, is shown. In panel (**b**), circles represent the samples from the first day of administration and diamonds represent the samples from the fifth day of administration. Clear clustering between the first and the fifth days and between the P and O samples is shown. In Panel (**c**), “1” stands for the first day and “5” the fifth day. The distance between the first- and fifth-day metabolomes is larger for the O samples, which shows the effect of OE administration. Furthermore, O samples from the fifth day merge with the P samples in a common cluster—a metabolome reset effect.

Subtracting the day-based effect Var_day_ and keeping as the discriminant variable the combination of the within-subject variation and the week-based effect (Var_within_ + Var_week_) (Figure 5a), a strong week-based effect was highlighted, where the samples from the first week of the administration (circles) were clustered separately from those of the second week (triangles) according to the first component (x-variate 1). Furthermore, in the second component (x-variate 2), strong clustering was also observed (Var_within_). This was probably due to subtle differences and variation in uncontrolled parameters such as sample handling and/or storage, differences in the environment/physiological situation of the subjects and a possible induced instability of the biomaterial used. The validation-tuning criteria presented maximum correlations of 0.96, 0.96 and 0.96 for each sPLS-DA dimension, when employing variable selection sizes of 50, 60 and 70 metabolites, respectively, for each dimension. When we kept Var_between_ as the discriminating value, nine clusters formed, showing that when taking the biological variation into account, it could be considered as the most influential factor of all.

Finally, it was essential to highlight the day-based effect by examining the two extremes of the administration days, i.e., the first vs. the fifth. We did so by subtracting the between-subject variation Var_between_ and keeping as discriminant variables the within-subject variation and the day-based effect (Var_within_ + Var_day_) (Figure 5b).

In this case, two main clusters could now be observed, demonstrating a systematic difference between the placebo (P) and oleuropein (O) samples, whereas separation could be also observed due to the day of administration. Figure 5b shows the difference between the first and fifth day of the administration for the O samples, and interestingly, a similar phenomenon was observed for the P samples. An elaborated version of Figure 5b is given in Figure 5c.

The day effect was notably smaller for the P compared to the O group (schematically, the length of the arrow between the centroids of the clusters was smaller in the case of P samples), denoting a marked effect of OE on the metabolome. Interestingly, according to the X-variate 1, the fifth day of OE administration was indistinguishable from the P group, a fact that further supports the assumption of a metabolome reset for samples from the fifth day of OE administration. Similar observations were obtained from the MVA of the remaining serum and urine datasets. Therefore, it was considered necessary to proceed with the evaluation of the loading plots, to discover possible biomarkers related to OE administration.

Overall, it is evident that simple MVA analysis is not sufficient for the full evaluation of such complex experiments since “sub-variances” contribute to the total variance, and the latter must be broken down mathematically. Thus, multilevel approaches should be employed to discern viable conclusions. In the current case, it seems that the remaining parts of the variation played a near-equally important role in the administration scheme, a suggestion that should be also studied further.

Maximum correlations of 0.98, 0.99 and 0.99 for the variable selection sizes of 50, 60 and 70 metabolites, respectively, for each sPLS-DA dimension were obtained by applying the validation-tuning criterion.

### 2.4. Metabolites

Two strategies were employed to extract the most influential loadings from the statistical analysis of all the datasets. According to the first strategy, the 10 most important loadings (largest absolute value) of the X-variate 1 were considered, followed by the 10 most important loadings of the X-variate 2 and consequently, on X-variate 3. The procedure stopped when all sparse components were selected and evaluated. The second strategy involved the selection of metabolites using adjustable-radius Euclidean distances to the ML-sPLS-DA variable plot, starting from 0.9 and decreasing using a 0.1 step. In other words, decreasing ellipse values were used and the variables between the Hotteling ellipse and the adjustable radius were selected.

The sparse loadings selected by either strategy are summarized in Table 1.

Additionally, the induced in-source fragmentation was analyzed by employing a deconvolution step using Mass Frontier 5.0.1 and its freely available counterpart AMDIS, producing possible pseudo-fragmentation spectra. Finally, the FDR (false discovery rate)-corrected results were evaluated to verify the significance of the up- or downregulated metabolites, and the results are tabulated in Tables SB2–SB4. The Metaboanalyst 5.0 platform was used for pathway analysis.

### 2.5. Biological Evaluation

OE administration increases the circulating acylcarnitines in serum, denoting elevated fatty acid catabolism, as it is well-known that acylcarnitines mediate their transfer into the mitochondria. The elevated levels of fatty acids in plasma result in their enhanced binding to serum albumin and thus the release of tryptophan. This could explain the finding of upregulated tryptophan metabolism found in O samples, where five metabolites out of 35 in total (M1–M5) contributing to this metabolic pathway could be identified.

Such an effect was observed in trained rats, where nutritional supplementation with fatty acids [49] resulted in increased concentrations of the so-called “free tryptophan” portion. This could, in turn, lead to elevated levels of the amino acid (tryptophan) in the brain, given that the blood-brain barrier is easily permeable for tryptophan. As tryptophan is an immediate precursor of serotonin, it can act positively on endurance and sensation of effort [50]. Thus, it was observed that the total exercise time was increased by 49.4% after receiving l-tryptophan versus the placebo in healthy sportsmen, as described in the literature [51]. The central fatigue phenomenon correlated with elevated l-tryptophan levels is difficult to be accounted for and could possibly be attributed to the enhanced training period that is induced by the activity of the amino acid. Closely related to serotonin is its main metabolite 5-hydroxy-indoleacetic acid, along its 5-methoxy analogue (M4) [52] degraded by monoamine oxidase (MAO), which often acts as a proxy for determining the parent molecule in urine [53]. Metabolite M4 is produced by the action of s-adenosyl-transferase as the methyl donor through acetylserotonin O-methyltransferase. The increased levels of the hydroxy-indoleacetic acid molecules are according to the increased levels of l-tryptophane, whereas it was found that high-intensity exercise increases its levels [54]. Meanwhile, M4 exhibited increased levels after exercise in transgenic mice with overexpressed PGC-1α [55]. Furthermore, M4 levels were found to be altered in dogs [56] and humans [57]. The microbial tryptophane degradation produces, among others, indoleacrylic acid (M2). The latter can activate the aryl hydrocarbon receptor (AhR), which in turn, can stimulate xenobiotics’ metabolism by inducing the cytochrome P450 enzyme system. Therefore, such mechanisms could possibly induce the fast degradation of OE, and ultimately, lead to the metabolome reset effect. The metabolite was also found in the sweat of exercising athletes [58]. Indole (M16) is another metabolite that is produced by the activity of gut microflora on tryptophan [59,60]. Furthermore, indole-based derivatives seem to play a major role in preventing oxidative effects in rats during exercise [61]. Tryptamine (M3) is formed by the catabolism of tryptophan by gut microbiota [62], and it was found that it acts on the parts of the brain releasing serotonin, enhancing the serotoninergic activity [63]. Therefore, it seems the proposed supplementation enhances the indole derivatives either through the human metabolism or by their production in the gut.

Phenylacetic acid (M17) is a product of phenylalanine metabolism. It was found that the urinary levels of the metabolite increase under strenuous exercise, which biotransforms, and therefore, reflects the amount of phenylethylamine [64]. The latter metabolite has strong antidepressant effects, and therefore, M17 is correlated with the euphoric effect of physical exercise and the therapeutic potential of the latter against depression [65]. Interestingly, 4-hydroxyphenyl acetic acid, a metabolite of M4, was shown to decrease during the intensive training of young athletes [66]. Estrone sulfate (M18) is the biotransformation product of estrone after its biotransformation by arylsulfatase. Although an estrogen, it was found that its levels increase after exercise, especially during the recovery period [67]. Sebacic (M19), i.e., 1,8-octane dicarboxylic acid, along with its counterpart 1,8-decane dicarboxylic acid, are considered as alternative energy sources in cases of type II diabetes or impairment of aerobic glycolysis [68]. It is postulated that in these cases, the medium-chain dicarboxylic acids cover part of the energy demands efficiently, reduce muscle fatigue and promote the completion of exercise [68]. Overall, consumption of OE-based herbal medicinal products could potentially boost the training capability.

## 3. Materials and Methods

### 3.1. Crossover Study Design

The study was carried out at Aristotle University of Thessaloniki, Greece. Nine young healthy males (A, B, C, D, E, F, G, H and I), physical education students in the age range of 20–22 years with no significant difference in the demographic characteristics (age, height, weight), participated in a double-blind crossover study. The participants received 1200 mg olive leaf extract enriched in OE or placebo, as nontransparent capsules, for one week in a randomized, balanced, double-blind manner. The extract contained 16–24% oleuropein and ≥30% olive phenols (10.2298/JSC0904367D). The participants provided blood and urine samples every morning before supplementation. During the first week of supplementation, the participants were requested to adhere to a given dietary plan and record their actual diets and physical activity. A two-week washout period followed, in which each participant received the alternate supplement for one week. During the second week of supplementation, each participant repeated the diet and physical activity recorded during the first week and was requested to follow a similar lifestyle pattern as in the first week. During the two first days of each week, the subjects received no supplementation, to provide samples as baseline measurements.

The protocol was approved by the Ethics Committee of the School of Physical Education and Sport Science of Aristotle University of Thessaloniki (approval number 107/2022) and was conducted according to the ICH-GCP guidelines (ICH GCP, 1996). All participants signed written informed consent to participate in the study.

### 3.2. Biofluid Collection

Urine samples for the measurements of 8-hydroxy-2′-deoxyguanosine (serving as an index of DNA damage [69]) and the metabolomic analysis were collected in appropriate urine collection vessels over 24 h after the supplementation, including a 12-h overnight fast. A gelatinized layer of metaphosphoric acid was applied to the vessels to inhibit bacterial growth. The urine samples were centrifuged, and aliquots were stored at −80 °C before use.

Blood samples were collected in vacutainer tubes. The serum and erythrocytes were separated immediately after the collection by centrifugation at 1800× *g* for 10 min at room temperature, and 500 μL aliquots of the supernatant were stored at −80 °C, until subsequent analysis. Serum samples were used for the analysis of the lipidemic profile (triglycerides, total cholesterol, HDL cholesterol and LDL cholesterol) and the metabolomic analysis, while erythrocytes were used to measure glutathione, the main antioxidant in the blood.

### 3.3. Samples for the Metabolomic Study

The participants received olive leaf extract (enriched in oleuropein or placebo) capsules, and the corresponding samples (serum and urine) were marked either as oleuropein-treated (O) or placebo (P), while the corresponding samples from the baseline period were marked as baseline (B). A total of 126 serum and 126 urine samples were collected and analyzed for the metabolomic study. The samples along with their corresponding labels are listed in Table SB5.

### 3.4. Chemicals, Reagents and Instrumentation

All solvents used in this study were of LC-MS grade. Acetonitrile (ACN), methanol (MeOH), water and formic acid were purchased from Honeywell Riedel-de Haën™ (Muskegon, MI, USA). Ultra-HPLC (UHPLC) analysis was performed by employing an Accela system (Thermo Scientific, Waltham, MA, USA) equipped with a binary pump, autosampler, online vacuum degasser and temperature-controlled column compartment. HRMS analysis was performed on a hybrid LTQ Orbitrap Discovery mass spectrometer (Thermo Scientific, Waltham, MA, USA). Centrifuging of the serum and urine samples was performed by a Mikro 200R centrifuge (Hettich Lab Technology, Tuttlingen, Germany), while evaporation was performed with the aid of a GeneVac HT-4X EZ-2 series evaporator Lyospeed ENABLED (GeneVac Ltd., Ipswich, UK).

### 3.5. Sample Pretreatment

A total of 126 serum samples were prepared following a modification of the large-scale metabolic profiling of serum samples [70]. The samples were allowed initially to thaw on ice at 4 °C for 30–60 min. Then, a 200 μL aliquot from each serum sample was placed into a labeled 1.5 mL Eppendorf tube, and 600 μL of MeOH was added, followed by vortexing for 15 s. The samples were subsequently centrifuged at 15,800× *g* for 15 min at room temperature to pellet the protein precipitate. Next, 185 μL aliquots from the supernatant were transferred into two separate labeled 1.5 mL Eppendorf tubes, and finally, evaporated (90 min at 50 °C). Consequently, 100 μL of water was added to each dry sample, which was vortexed for 15 s, centrifuged at 15,800× *g* for 15 min and transferred to a 200 μL insert. The inserts were placed in appropriate screw-capped autosampler vials.

Urine samples were prepared following a common metabolic profiling urine protocol [71]. Briefly, 60 μL of each urine sample was centrifuged at 10,000× *g* for 10 min at 4 °C to remove particulates, and 50 μL from each sample was transferred to 200 μL inserts placed in appropriate screw-capped autosampler vials with the addition of 100 μL water. The mixture was briefly vortexed for 30 s.

### 3.6. Biochemical Analyses

A series of biochemical analyses were also conducted to discern the effect of OE supplementation on the redox status of the subjects. To this end, measurements of glutathione were carried out using erythrocytes, followed by analysis of the lipidemic profile (triglycerides, total cholesterol, HDL cholesterol, LDL cholesterol) by photometric methods, to measure the redox status (urate, bilirubin, malondialdehyde, protein carbonyls) and 8-hydroxy-2′-deoxyguanosine (an index of DNA damage) in athletes’ urine. Data were analyzed by two-way ANOVA with repeated measures on both factors. All biochemical analyses were conducted at the School of Physical Education and Sports Science at Aristotle University of Thessaloniki (Thessaloniki, Greece) by the team of Professor V. Mougios.

### 3.7. UHPLC-HRMS Analysis

#### 3.7.1. Serum Samples’ Acquisition

An ACQUITY UPLC BEH C_18_ (2.1 × 100 mm, 1.7 μm) reversed-phase column (Waters Corp., Milford, MA, USA) preceded by a precolumn (Waters VanGuard 5 × 2.1 mm, 1.7 μm) was used for the chromatographic separation. The mobile phase consisted of solvents A) aq. formic acid, 0.1% (*v*/*v*), and B) MeOH formic acid, 0.1% (*v*/*v*).

The LC/MS methodologies for the serum analysis in the positive and negative ion modes can be found in Table SC1 [70].

#### 3.7.2. Urine Samples’ Acquisition

An ACQUITY UPLC BEH C_18_ (2.1 × 100 mm, 1.7 μm) reversed-phase column (Waters Corp., Milford, MA, USA) preceded by a precolumn (Waters VanGuard 5 × 2.1 mm, 1.7 μm) was also used for the chromatographic separation of the urine samples. The mobile phase consisted of solvents (A) aq. formic acid, 0.1% (*v*/*v*), and (B) ACN formic acid, 0.1% (*v*/*v*).

The LC/MS methodologies for the serum analysis in the positive and negative ion modes can be found in Table SC2.

#### 3.7.3. Quality Control Samples

Serum and urine pooled samples were used as quality control (QC) samples. The QC samples were created by pooling every plasma or urine sample, respectively. For both studies, five QC samples were injected at the beginning of each analytical batch, with one QC every 10 samples throughout the run and five QC samples at the end of the batch, while the ion transfer tube was removed and cleaned every 50 injections.

#### 3.7.4. Mass Spectrometry Data Processing

The software used for peak picking were Xcalibur^®^ (Thermo Fisher Scientific) and MZmine 2.10 (http://mzmine.sourceforge.net, accessed on 28 December 2021). The matrix preparation for statistical analysis was done using Microsoft Excel 2012. The xMSanalyzer package (https://sourceforge.net/projects/xmsanalyzer/, accessed on 28 December 2021) implemented with R statistical language 3.0.2 (http://cran.r-project.org, accessed on 28 December 2021) was employed for feature annotation and Metaboanalyst 5.0 (http://www.metaboanalyst.ca, accessed on 28 December 2021) for pathway analysis. For pre-processing of the MS data using QCs, the QC-RLSC algorithm was employed as implemented for the MetMSLine scripts (https://github.com/WMBEdmands/MetMSLine_Scripts, accessed on 28 December 2021). Details can be found in Supplementary Methodologies B,C.

#### 3.7.5. Statistical Data Analysis

MVA data analyses, including PCA, PLS-DA and OPLS-DA, were performed using SIMCA P+ 10.5 (Umetrics, Umea, Sweden), EZinfo (Umetrics, Umea, Sweden) and Metaboanalyst. Kernel-based MVA methods were also employed (e.g., kPCA) using a variety of kernels such as polynomial Gaussian, etc., via the kernlab R package. Sparse principal component analysis (sPCA), sparse independent principal component analysis (siPCA), sparse partial least squares–discriminant analysis (sPLS-DA) and multilevel sparse partial least squares-discriminant analysis (ML-sPLS-DA) were performed using the mixOmics package (https://bioconductor.org/packages/release/bioc/html/mixOmics.html, accessed on 28 December 2021) as implemented in R 3.0.2 (http://http://cran.r-project.org, accessed on 28 December 2021). ANOVA was performed on the important metabolites and their intensity values selected as biomarkers by the ML-sPLS-DA models. The statistical procedures we employed are described in Supplementary Methodology C, except for ML-sPLSDA, which was the methodology that provided the statistically meaningful results.

#### 3.7.6. Multilevel Sparse Partial Least Squares–Discriminant Analysis

ML-sPLS-DA is an MVA method used for the pairwise comparison of different groups in crossover studies, where each subject becomes its own control in a random order [40]. ML-sPLS-DA is considered the method of choice in human metabolomics interventional studies since the inter-individual variability caused by genetic, dietary, lifestyle and environmental factors can obscure nutrition-related metabolic effects. In our work, ML-sPLS-DA was performed using the mixOmics package implemented with the freely available R statistical language 3.0.2. The quality of the ML-PLS-DA models was judged by the goodness-of-fit (R^2^) and the predictive ability (Q^2^) parameter, calculated by internal seven-fold cross-validation and the leave-out methodology.

#### 3.7.7. Variable Selection—Variable Importance in the Projection

The variable importance in the projected (VIP) values of each variable were calculated as indicators of their contribution to the sample classification. Variables with a VIP value > 1.5 were considered important in discriminating between groups.

#### 3.7.8. Sparse Loadings

The first 50 sparse loadings as obtained from the ML-sPLS-DA models were considered important in discriminating between groups and were selected for further evaluation. A plot containing the selected sparse variables was constructed, using the “PlotVar” command and by applying a “rad.in” value of 0.9.

## 4. Conclusions

In the present study, the integrated mass spectrometry-based metabolomic approach consisted of two steps: an ML and an MVA approach. These were utilized to analyze serum and urine samples from a double-blind crossover-designed study, encompassing the administration an olive leaf extract enriched in OE to young, healthy males. The ML approach allowed the deconvolution of the between-subject variation, which was separated from the within-subject variation, while simultaneously taking into account the other types of variation, such as that caused by the week or from the day of administration. Using this approach, 29 differentially expressed metabolites were identified, revealing pathway-specific expression profiles.

Overall, supplementation with an olive leaf extract enriched in OE was found to alter the serum and urine metabolomes of the athletes compared to the placebo administration, demonstrating that OE could have an impact on training. Importantly, the findings support the notion that OE administration alters an array of factors concerning crucial biochemical pathways that are implicated in physical activity. In this research, the metabolism of tryptophan was found to be upregulated after OE supplementation and increased the circulating acylcarnitines in the serum and urine.

## Figures and Tables

**Table 1:** Candidate metabolites identified in O and P groups from serum- and urine-based metabolomic approaches obtained from both (+) and (–) ESI analyses. The features presented are the (a) metabolite ID, (b) *m*/*z* feature, (c) tR (min), (d) complex/dimer/product/precursor, (e) experimental deconvoluted *m*/*z* (precursor), (f) theoretical *m*/*z* (precursor), (g) Δm (ppm), (h) corresponding RDB value, (i) deconvoluted pseudo-MS/MS ions, (j) possible identification name,(k) possible molecular formula, (l) corresponding monoisotopic exact mass, (m) change trend in O group compared with P group (up- or downregulation), (n) fold change and (o) corresponding dataset (serum or urine, positive or negative ESI dataset).

ID	*m*/*z*	t_R_ (min)	Complex/ Dimer/ Product/ Precursor	Experimental Deconvoluted *m*/*z* (Precursor)	Theoretical *m*/*z* (Precursor)	Δm (ppm)	RDB	Pseudo-MS/MS ions	Putative Identification	Molecular Formula	Exact Mass	Trend	Fold Change +/− SD	Dataset
M1	188.0704	1.02	Product	205.0970	205.0972	−0.9	6.5	-	L-tryptophan	C_11_H_12_N_2_O_2_	204.0899	↑ ^a^	2.6 (0.4)	Urine ESI(+)
M2	188.0704	1.46	Precursor	188.0704	188.0703	−1.0	7.5	-	Indoleacrylic acid	C_11_H_9_NO_2_	187.1947	↑	1.9 (0.5)	Urine ESI(+)
M3	161.1070	3.96	Precursor	161.1070	161.1073	−1.9	5.5	-	Tryptamine	C_10_H_12_NO_2_	160.2157	↑	1.9 (0.3)	Urine ESI(+)
M4	206.0810	4.88	Precursor	206.0810	206.0812	0.2	6.5	-	5-methoxy-indolelactic acid	C_11_H_11_NO_3_	205.0738	↑	1.9 (0.3)	Urine ESI(+)
M5	221.1534	4.77	Product	286.2013	286.2013	0	2.5	-	2-octenoylcarnitine	C_15_H_27_NO_4_	285.3792	↑	1.6 (0.2)	Urine ESI(+)
M6	279.1338	3.02	Precursor	279.1338	279.1339	−0.5	6.5	-	L-phenylalanyl-L-hydroxyproline	C_14_H_18_N_2_O_4_	278.3037	↑	1.1 (0.3)	Urine ESI(+)
M7	316.2481	5.67	Precursor	316.2481	316.2482	−0.4	1.5	-	Decanoylcarnitine	C_17_H_33_NO_4_	315.4482	↑	1.6 (0.3)	Urine ESI(+)
M8	251.1277	5.80	Precursor	251.1277	251.1278	–0.3	5.5	-	Ubiquinone-1	C_14_H_18_O_4_	250.2903	↑	1.1 (0.2)	Urine ESI(+)
M9	314.2325	5.30	Precursor	314.2325	314.2326	−0.4	2.5	-	9-decenoylcarnitine	C_17_H_31_NO_4_	313.4323	↑	1.5 (0.4)	Urine ESI(+)
M10	302.2325	5.23	Precursor	302.2325	302.2326	−0.6	1.5	-	2.6 dimethylheptanoyl carnitine	C_16_H_31_NO_4_	301.4216	↑	1.6 (0.3)	Urine ESI(+)
M11	130.0494	4.30	Product	147.0760	147.0764	−2.7	1.5	-	L-glutamine	C_5_H_10_N_2_O_3_	146.1445	↓		Urine ESI(+)
M12	293.1471	4.30	Dimer (2M + H)					-					1.1 (0.4)	
M13	147.0760	4.30	Precursor					-						
M14	286.2012	4.55	Precursor	286.2012	286.2013	−0.5	2.5	-	2-octenoylcarnitine	C_15_H_27_NO_4_	285.3792	↓	1.7 (0.3)	Urine ESI(+)
M15	330.2274	4.24	Precursor	330.2274	330.2275	−0.6	2.5	-	6-keto-decanoylcarnitine	C_17_H_31_NO_5_	329.4317	↑	1.6 (0.4)	Urine ESI(+)
M16	105.0329	1.71	Product	118.0646	118.0651	−4.6	5.5	-	Indole	C_8_H_8_N	117.1479	↓	1.9 (0.3)	Urine ESI(+)
M17	137.0593	4.49	Precursor	137.0593	137.0597	−3	4.5		Phenylacetic acid	C_8_H_8_O_2_	136.0524	↑	1.8 (0.3)	Urine ESI(+)
M18	349.1121	2.96	Precursor	349.1121	349.1104	4.8	8.5	215/183/171/157/133	Estrone-sulfate	C_18_H_22_O_5_S	350.4290	↑	1.8 (0.2)	Urine ESI(−)
M19	201.1134	3.58	Precursor	201.1134	201.1121	6.2	2.5	183/157	Sebacic acid	C_10_H_18_O_4_	202.2475	↓	1.6 (0.3)	Urine ESI(−)
M20	389.0989	0.90	Dimer (2M + H)	194.0452	194.0448	2.1	6.5	178/96	Hydroxyhippuric acid	C_9_H_9_NO_4_	195.1721	↓	1.2 (0.4)	Urine ESI(−)
M21	357.1092	1.71	Dimer (2M + H)	178.0501	178.0499	1.3	6.5	134/96	Hippuric acid	C_9_H_9_NO_3_	179.1727	↑	1.2 (0.4)	Urine ESI(−)
M22	758.5666	20.1	Precursor	758.5666	758.5694	−3.7	3.5	-	Glycerophospholipid Skeleton	C_42_H_80_NO_8_P	757.5621	↑	1.1 (0.2)	Serum ESI(+)
M23	158.0806	10.4	Precursor	158.0806	158.0812	−3.1	2.5	79/80/97/98/99	Tiglylglycine	C_7_H_11_NO_3_	157.1671	↓	1.1 (0.4)	Serum ESI(+)
M24	414.2993	10.4	Precursor	414.2993	414.3003	−2.3	7.5	-	N-docosahexaenoyl GABA	C_26_H_39_NO_3_	413.2924	↓	1.3 (0.2)	Serum ESI(+)
M25	465.3042	24.1	Product	465.3042	465.3033	1.9	5.5	97/385	Cholesterol sulfate	C_27_H_46_O_4_S	466.7170	↓	1.3 (0.3)	Serum ESI(−)
M26	167.0214	0.9	Product	167.0214	167.0200	6.2	6.5	96/124	Uric acid	C_5_H_5_O_3_N_4_	168.1103	↓	1.1 (0.3)	Serum ESI(−)
M27	391.2853	9.9	Product	391.2853	391.2843	1.2	5.5	–	Bile acid	C_24_H_40_O_4_	392.2926	↓	1.1 (0.3)	Serum ESI(−)
M28	103.0399	1.1	Product	103.0399	103.0390	3.7	1.5	–	Hydroxybutyric acid	C_4_H_8_O_3_	104.1045	↓	1.2 (0.4)	Serum ESI(−)
M29	135.0302	1.0	Product	135.0302	135.0288	6.1	1.5	–	Erythronic acid/Threonic acid	C_4_H_8_O_5_	136.1033	↓	1.3 (0.2)	Serum ESI(−)

^a^ (↑): upregulated in O group; (↓): downregulated in O group.

## Data Availability

The data presented in this study are available in this article and supplementary material.

## Supplementary Materials

The following supporting information are available online at https://www.mdpi.com/article/10.3390/metabo12020195/s1: **Supplementary Figures.** Figure SA1, Venn diagram from the two tested algorithms—“baseline cut-off” (A) and “wavelets” (B)—with 887 features in the common section, Figure SA2, PCA score plots of representative individuals’ (B, C, H and F) urine samples (P and O) after UV scaling, obtained from UHPLC-(+)ESI-HRMS analysis. A clear trend toward differentiation between P (red) and O (black) samples was observed for each individual, Figure SA3, PLS-DA score plots (UV scaled) of B, P and O urine samples from all the days of administration, obtained by UHPLC-(+)ESI-HRMS. No significant clustering was observed between the B (green points), O (black points) and P samples (red points), Figure SA4, PLS-DA score plot (UV scaled) of P and O urine samples from all the days of administration, obtained by UHPLC-(+)ESI-HRMS. No significant clustering was observed between the O (black points) and P samples (red points), Figure SA5, OPLS-DA score plot (UV scaled) of P and O urine samples from all the days of administration, obtained by UHPLC-(+)ESI-HRMS. No significant clustering was observed between the O (black points) and P samples (red points), even if they tend to form two separate classes/groups, Figure SA6, Two-level ML-PLS-DA analysis of the P and O urine samples from all the days of administration, obtained by UHPLC-(+)ESI-HRMS. The 3D multilevel PLS-DA score plot (Comp 1, Comp 2, Comp 3) of the combination of within-subject variation and day-based effect (Var_within_ + Var_day_), with the biological variation (Var_between_) subtracted. Red circles represent the P samples. Black circles represent the O samples. Clear clustering between P and O samples is shown. **Supplementary Tables.** Table SB1, A list of the tested samples along with their corresponding labels, which we used to examine each individual. Oleuropein samples (O) are in black and placebo (P) samples are in red. Baseline samples (Days “−1” and “0”) (light grey) were not considered in this step of MVA, Table SB2, ANOVA results for the significant metabolite M1, Table SB3, ANOVA results for the significant metabolite M5, Table SB4, ANOVA results for the significant metabolite M19, Table SB5, A list of the tested samples from the crossover study along with their corresponding labels. Baseline samples (B) are in green, oleuropein samples (O) are in black and placebo (P) samples are in red. **Supplementary Methodologies.** *(A) UHPLC-HRMS Methodologies* Table SC1, Gradient elution programs and MS parameters applied for the UHPLC-HRMS analysis of the serum samples in (+) and (−) ESI modes, Table SC2, Gradient elution program and MS parameters applied for the UHPLC-HRMS analysis of the urine samples in (+) and (−) ESI modes; *(B) Mass Spectrometry Data Processing* Peak picking, data pre-processing, feature annotation and pathway analysis procedures; *(C) Statistical Data Analysis*.
