# Supplementary material for: Effect of Supplementation with Olive Leaf Extract Enriched with Oleuropein on the Metabolome and Redox Status of Athletes’ Blood and Urine—A Metabolomic Approach
Source: Metabolites. 2022 Feb 20;12(2):195. doi: 10.3390/metabo12020195 (PMC8878006; doi:10.3390/metabo12020195)
Supplement: Supplementary file 1 [file metabolites-12-00195-s001.zip › Supplementary SA. Figures.pdf]

Supplementary SA. Figures

Contents

Figure SA1. 2
Figure SA2. 2
Figure SA3. 3
Figure SA4. 3
Figure SA5. 4
Figure SA6. 4

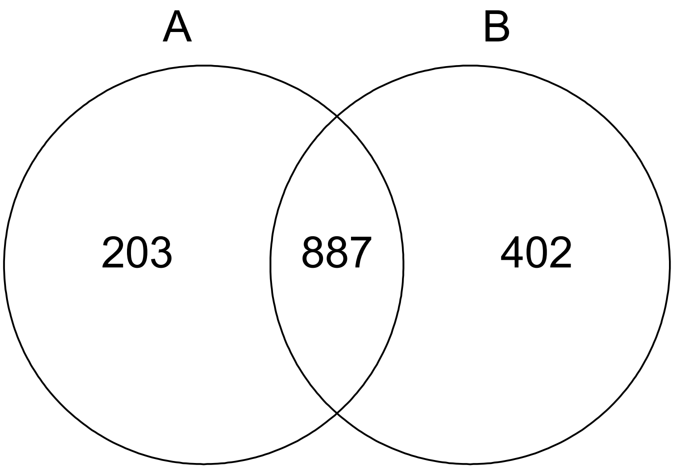

Figure SA1. Venn diagram from the two tested algorithms; “baseline cut-off” (**A**) and “Wavelets” (**B**) 887 common features are in the common section.

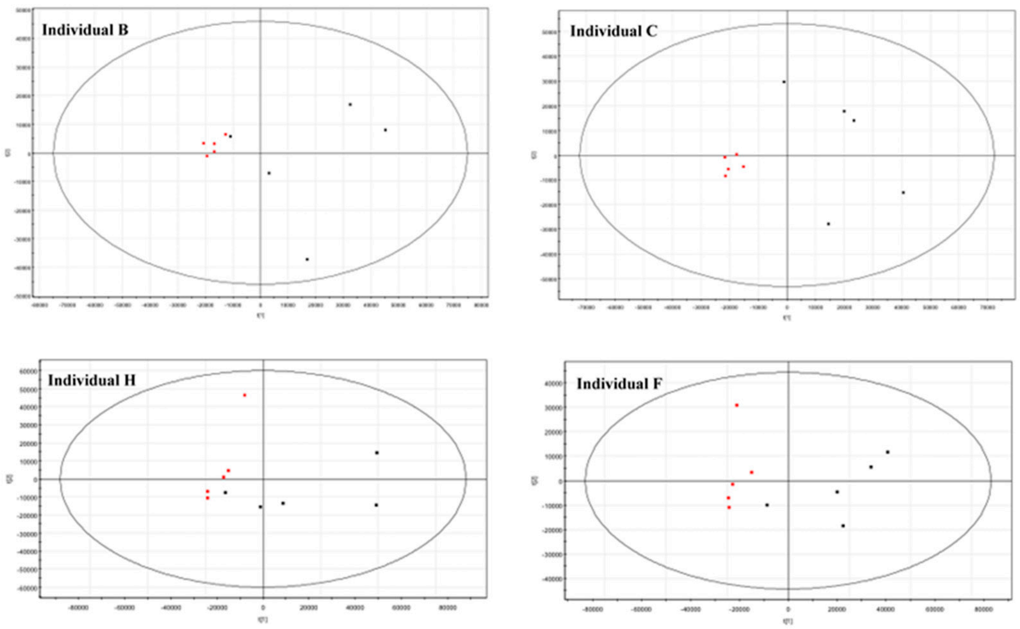

Figure SA2. PCA scores plots of representative individuals’ (B, C, H and F) urine samples (P and O) after UV scaling, obtained from UHPLC-(+)ESI-HRMS analysis. A clear trend towards the differentiation between P (red) and O (black) samples was observed in each individual separately.

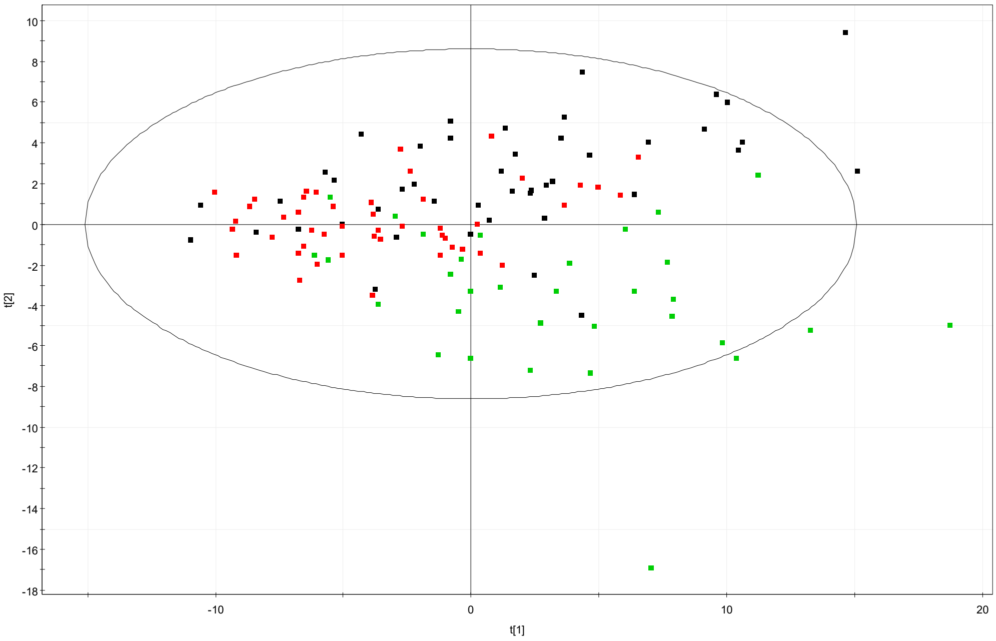

Figure SA3. PLS-DA scores plot (UV scaled) of B, P and O urine samples from all the days of the administration, obtained by UHPLC-(+)ESI-HRMS. No significant clustering was observed between the B samples (green points) the O samples (black points) and the P samples (red points).

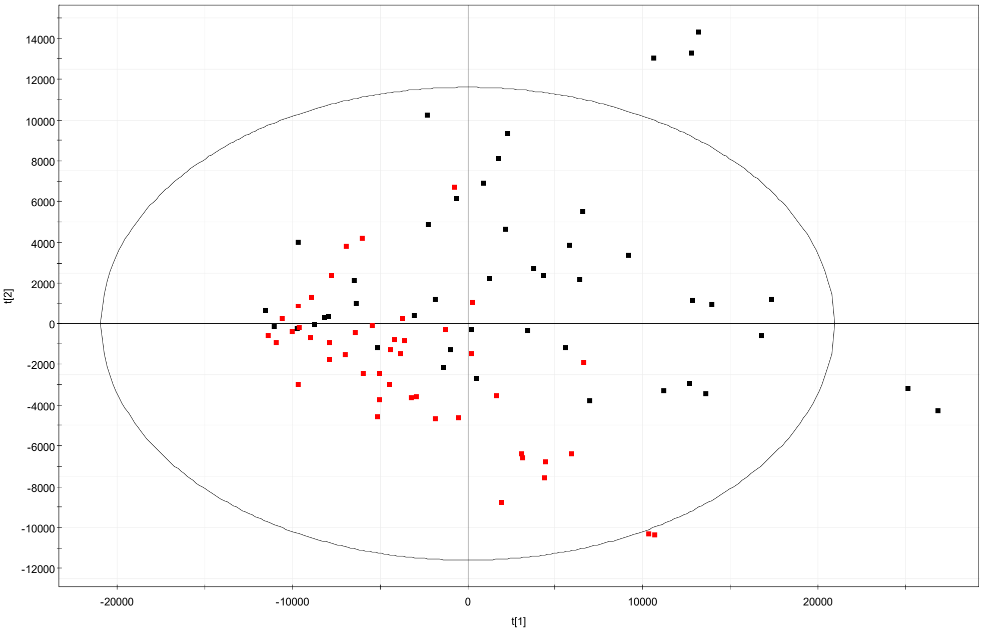

Figure SA4. PLS-DA scores plot (UV scaled) of P and O urine samples from all the days of the administration, obtained by UHPLC-(+)ESI-HRMS. No significant clustering was observed between the O samples (black points) and the P samples (red points).

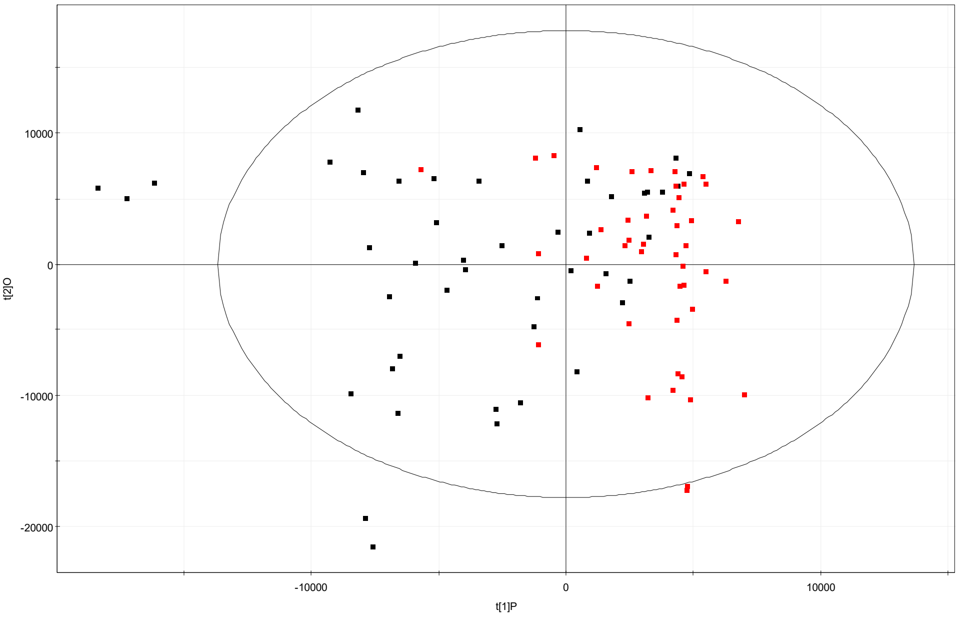

Figure SA5. OPLS-DA scores plot (UV scaled) of P and O urine samples from all the days of the administration, obtained by UHPLC-(+)ESI-HRMS. No significant clustering was observed between the O samples (black points) and the P samples (red points), even if they have the tendency to form two separate classes/groups.

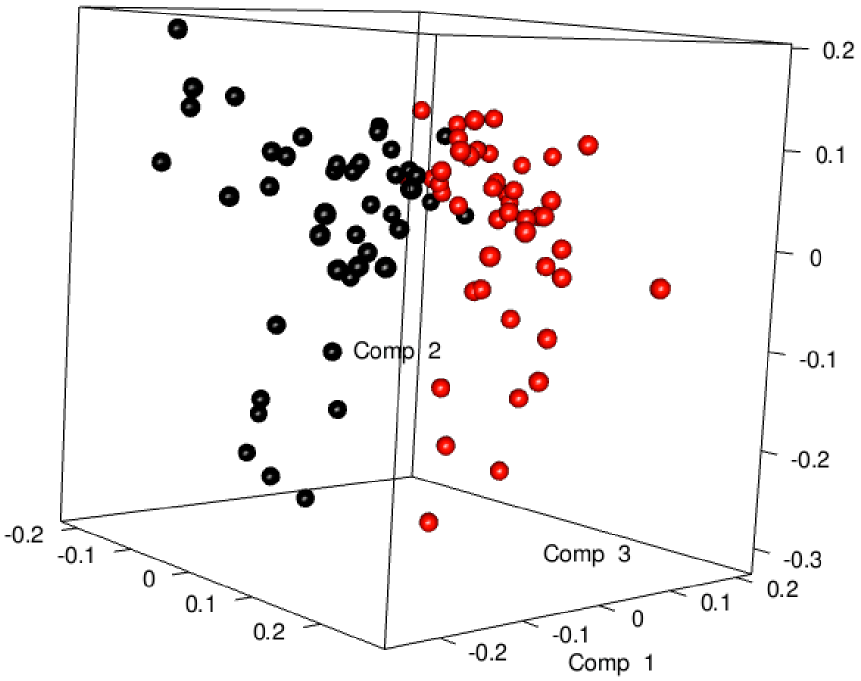

Figure SA6. 2-level ML-PLS-DA analysis of the P and O urine samples from all the days of administration, obtained by UHPLC-(+)ESI-HRMS. The 3-D multilevel PLS-DA scores plot (Comp 1, Comp 2, Comp 3) of the combination of the within subject variation and the day-based effect (Var_{within} + Var_{day}) with the biological variation ($Var_{between}$) being subtracted is shown. Red circles represent the P samples. Black circles represent the O samples. A clear clustering between P and O samples, is shown.
