# Supplementary material for: Effect of Supplementation with Olive Leaf Extract Enriched with Oleuropein on the Metabolome and Redox Status of Athletes’ Blood and Urine—A Metabolomic Approach
Source: Metabolites. 2022 Feb 20;12(2):195. doi: 10.3390/metabo12020195 (PMC8878006; doi:10.3390/metabo12020195)
Supplement: Supplementary file 1 [file metabolites-12-00195-s001.zip › Supplementary SB. Tables.pdf]

Supplementary SB. Tables

Contents

Table SB1 2

Table SB2 3

Table SB3 3

Table SB4. 3

Table SB5 4

Table SB1. A list of the tested samples along with their corresponding labels, used for the examination of each individual separately. Oleuropein samples (O) are in black and placebo (P) samples are in red. Baseline samples (Days "-1" and "0") (light grey) were not considered in this step of MVA.

Weeks		1st week							2nd week						
		Days							Days						
Subjects	Days	-1	0	1	2	3	4	5	-1	0	1	2	3	4	5
A		1	11	21	31	41	51	61	1B	11B	21B	31B	41B	51B	61B

Weeks		1st week							2nd week						
Subjects	Days	-1	0	1	2	3	4	5	-1	0	1	2	3	4	5
B		3	13	23	33	43	53	63	3B	13B	23B	33B	43B	53B	63B

Weeks		1st week							2nd week						
Subjects	Days	-1	0	1	2	3	4	5	-1	0	1	2	3	4	5
C		4	14	24	34	44	54	64	4B	14B	24B	34B	44B	54B	64B

Weeks		1st week							2nd week						
Subjects	Days	-1	0	1	2	3	4	5	-1	0	1	2	3	4	5
D		5	15	25	35	45	55	65	5B	15B	25B	35B	45B	55B	65B

Weeks		1st week							2nd week						
Subjects	Days	-1	0	1	2	3	4	5	-1	0	1	2	3	4	5
E		6	16	26	36	46	56	66	6B	16B	26B	36B	46B	56B	66B

Weeks		1st week							2nd week						
Subjects	Days	-1	0	1	2	3	4	5	-1	0	1	2	3	4	5
F		7	17	27	37	47	57	67	7B	17B	27B	37B	47B	57B	67B

Weeks		1st week							2nd week						
Subjects	Days	-1	0	1	2	3	4	5	-1	0	1	2	3	4	5
G		8	18	28	38	48	58	68	8B	18B	28B	38B	48B	58B	68B

Weeks		1st week							2nd week						
Subjects	Days	-1	0	1	2	3	4	5	-1	0	1	2	3	4	5
H		9	19	29	39	49	59	69	9B	19B	29B	39B	49B	59B	69B

Weeks		1st week							2nd week						
Subjects	Days	-1	0	1	2	3	4	5	-1	0	1	2	3	4	5
I		10	20	30	40	50	60	70	10B	20B	30B	40B	50B	60B	70B

Table SB2. ANOVA results for the significant metabolite M1.

**Analysis of Variance (One-Way) Metabolite M1**						
**Summary**						
Groups	*Sample size*	*Sum*	*Mean*	*Variance*		
O	18	15558222.91	864345.71722	3.73796E+11		
P	18	7690860.74	427270.04111	1.58347E+11		
**ANOVA**						
Source of Variation	*SS*	*df*	*MS*	*F*	*p-level*	*F crit*
Between Groups	1.71932E+12	1	1.71932E+12	6.46186	0.01575	4.13002
Within Groups	9.04643E+12	34	2.66071E+11			
Total	1.07657E+13	35				

Table SB3. ANOVA results for the significant metabolite M5.

**Analysis of Variance (One-Way) Metabolite M5**						
**Summary**						
Groups	*Sample size*	*Sum*	*Mean*	*Variance*		
O	18	6153587.153	341865.95294	1.68643E+11		
P	18	2147063.846	119281.32478	3.20009E+10		
**ANOVA**						
Source of Variation	*SS*	*df*	*MS*	*F*	*p-level*	*F crit*
Between Groups	4.45895E+11	1	4.45895E+11	4.44463	0.0426	4.13002
Within Groups	3.41095E+12	34	1.00322E+11			
Total	3.85685E+12	35				

Table SB4. ANOVA results for the significant metabolite M19.

**Analysis of Variance (One-Way) Metabolite M19**						
**Summary**						
Groups	*Sample size*	*Sum*	*Mean*	*Variance*		
O	18	33925758.693	1884764.37183	1.02931E+13		
P	18	623969.718	34664.98433	1016438443.1 5771		
**ANOVA**						
Source of Variation	*SS*	*df*	*MS*	*F*	*p- level*	*F crit*

Between Groups	3.08058E+13	1	3.08058E+13	5.98514	0.01975	4.1302
Within Groups	1.75E+14	34	5.14705E+12			
Total	2.05805E+14	35				

Table SB5. A list of the tested samples from the crossover study along with their corresponding labels. Baseline samples (B) are in green, oleuropein samples (O) are in black and placebo (P) samples are in red.

Weeks	1st week							2 weeks wash out	2nd week						
Subjects / Days	-1	0	1	2	3	4	5		-1	0	1	2	3	4	5
A	1	11	21	31	41	51	61		1B	11B	21B	31B	41B	51B	61B
B	3	13	23	33	43	53	63		3B	13B	23B	33B	43B	53B	63B
C	4	14	24	34	44	54	64		4B	14B	24B	34B	44B	54B	64B
D	5	15	25	35	45	55	65		5B	15B	25B	35B	45B	55B	65B
E	6	16	26	36	46	56	66		6B	16B	26B	36B	46B	56B	66B
F	7	17	27	37	47	57	67		7B	17B	27B	37B	47B	57B	67B
G	8	18	28	38	48	58	68		8B	18B	28B	38B	48B	58B	68B
H	9	19	29	39	49	59	69		9B	19B	29B	39B	49B	59B	69B
I	10	20	30	40	50	60	70		10B	20B	30B	40B	50B	60B	70B
