# Supplementary material for: Effect of Supplementation with Olive Leaf Extract Enriched with Oleuropein on the Metabolome and Redox Status of Athletes’ Blood and Urine—A Metabolomic Approach
Source: Metabolites. 2022 Feb 20;12(2):195. doi: 10.3390/metabo12020195 (PMC8878006; doi:10.3390/metabo12020195)
Supplement: Supplementary file 1 [file metabolites-12-00195-s001.zip › Supplementary SC. Methodologies.pdf]

Supplementary SC. Methodologies

Contents

A. UHPLC-HRMS Methodologies 2
Table SC1 2
Table SC2 2
B. Mass Spectrometry Data processing (Peak picking, data pre-processing, feature annotation and pathway analysis procedures) 3
Peak picking procedures 3
MZmine process. 3
Microsoft Excel process. 6
xMSanalyzer 6
MetMSLine process. 6
MetaboAnalyst process. 6
Metabolic pathway analysis 7
Identification and structural elucidation of potential target metabolites. 7
C. Statistical Data analysis 8
Principal component analysis. 8
Sparse principal component analysis 8
Partial least squares–discriminant analysis. 8
Orthogonal partial least squares–discriminant analysis. 8
Sparse partial least squares–discriminant analysis. 8

A. UHPLC-HRMS Methodologies

Table SC1. Gradient elution programs and MS parameters applied for the UHPLC-HRMS analysis of the serum samples in (+) and (-) ESI modes.

**UHPLC parameters**			
Column: ACQUITY UPLC BEH C_{18} (2.1 × 100 mm, 1.7 μm) reversed phase			
Mobile phase: aq. formic acid, 0.1% (*v/v*) (A) and methanol formic acid, 0.1% (*v/v*) (B)			
Flow rate: 0.36 mL min^{-1} ESI (+) / 0.40 mL min^{-1} ESI (-)			
Column temperature: 50 °C			
Tray temperature: 4 °C			
Injection volume: 10 μL			
ESI (+)	Gradient Program		
	Time (min)	A%	B%
	0	100	0
	1	100	0
	16	0	100
	20	0	100
	22	100	0
	24	100	0
ESI (-)	Gradient Program		
	Time (min)	A%	B%
	0	100	0
	2	100	0
	17	0	100
	22	0	100
	24	100	0
	26	100	0
**HRMS parameters**			
30000 resolution			
Centroid mode			
ESI (+)	Capillary temperature (°C): 356		
	Capillary voltage (V): -60		
	Tube lens (V): 110		
	Spray voltage (kV): 3.50		
	Sheath gas flow (arb. units): 30		
	Aux gas flow (arb. units): 10		
ESI (-)	Capillary temperature (°C): 356		
	Capillary voltage (V): 20		
	Tube lens (V): -49		
	Spray voltage (kV): 3.10		
	Sheath gas flow (arb. units): 30		
	Aux gas flow (arb. units): 10		

Table SC2. Gradient elution program and MS parameters applied for the UHPLC-HRMS analysis of the urine samples in (+) and (-) ESI modes.

**UHPLC parameters**
Column: ACQUITY UPLC BEH C_{18} (2.1 × 100 mm, 1.7 μm) reversed phase

Mobile phase: aq. formic acid, 0.1% (*v/v*) (A) and ACN formic acid, 0.1% (*v/v*) (B)<br>Flow rate: 0.5 mL min^{-1}<br>Column temperature: 50 °C<br>Tray temperature: 4 °C<br>Injection volume: 10 μL			
	Gradient Program		
	Time (min)	A%	B%
	0	99	1
	1	99	1
	3	85	15
	6	50	50
	9	5	95
	10	5	95
	10.1	99	1
	12	99	1
**HRMS parameters**<br>30000 resolution<br>Centroid mode			
ESI (+)	Capillary temperature (°C): 356<br>Capillary voltage (V): -60<br>Tube lens (V): 110<br>Spray voltage (kV): 3.50<br>Sheath gas flow (arb. units): 30<br>Aux gas flow (arb. units): 10		
ESI (-)	Capillary temperature (°C): 356<br>Capillary voltage (V): 20<br>Tube lens (V): -49<br>Spray voltage (kV): 3.10<br>Sheath gas flow (arb. units): 30<br>Aux gas flow (arb. units): 10		

B. Mass Spectrometry Data processing (Peak picking, data pre-processing, feature annotation and pathway analysis procedures)

Peak picking procedures.
Initially, Xcalibur® was used to convert the instrument native Xcalibur data files (*.raw) to the general and more exchangeable cdf data format (*.cdf) with the embedded to the software Xconvert program. Data were then imported to MZmine 2.10 and processed applying the peak detection, deconvolution, normalization, deisotoping, alignment and gap filling procedures.

MZmine process.
Initially, the serum data set from ESI (+) acquisition was imported as centroid LC–HRMS individual files (*.cdf) in MZmine and appropriately filtered to generate corresponding new data files with corrected baseline intensities. In detail, the baseline correction was performed using the base peak intensity chromatogram type and applying a value of 500 for the smoothing, 0.001 for the asymmetry and a value of 1 for the m/z bin width. Then, a list of ions for each scan in the baseline-corrected data file was generated for every data file using the centroid mass detection module and applying the appropriate level for the elimination of noise, in this case 1E4 counts per second (cps). The centroid module is suitable for already centroided data, while the noise level is considered as the minimum intensity of a data point to be considered as part of a chromatogram. All data points below this intensity

level were ignored. Subsequently, the chromatogram builder algorithm has been applied, where the mass lists generated for each MS scan in the previous procedure were used in order to build a chromatogram for each mass that could be detected continuously over a predetermined number of scans (defined in terms of peak width). This step was carried out applying the following parameters: 0.05 min minimum time spam, 1E4 minimum height and 5 ppm *m/z* tolerance. Following, the generated chromatograms were deconvoluted into individual peaks by the Wavelets (XCMS) deconvolution algorithm setting the signal/noise threshold to 5, the wavelet scales from 0.1 to 5 min and the peak duration range from 0.1 to 1 min. To avoid duplicating the peaks due to the presence of their isotopic pattern, the deconvoluted data were processed using the isotopic peak grouper algorithm, which removed the additional isotopic peaks from the peak list. To accomplish this operation, the *m/z* tolerance was set to 5 ppm, the t_R tolerance to 0.05 min whereas only single charged ions were considered. After this step, possible adducts or peak complexes were recognized using the identification of adducts and peak complexes module and consequently unwanted systematic bias between measurements was removed applying the normalization module. The corresponding peaks from the B, P and O serum samples, which were analyzed in (+) ESI mode, were compared using the random sample consensus (RANSAC) aligner algorithm, since it provides the best results among all the tested algorithms[51]. The purpose of peak list alignment was to match relevant peaks in terms of t_R and *m/z* values across multiple samples and to generate a new aligned peak list. The parameters used in this step were 5 ppm *m/z* tolerance, 0.03 min t_R tolerance and 0.05 min t_R tolerance after correction, 15000 RANSAC iterations, 20% minimum number of points, a threshold value of 4 employing a non-linear model. Following alignment, the resulting peak list may contain missing peaks as a product of deficient peak detection or a mistake in the alignment of different peak lists. The fact that one peak is missing after the alignment does not imply that the peak does not exist but rather in most cases it is present but was undetected by the previous algorithms. Thus, the peak finder algorithm was used to fill the gaps in the peak list when it is possible according with the following parameters: 80% intensity tolerance, 5 ppm *m/z* tolerance and 0.05 min t_R tolerance, where the *m/z* and the t_R tolerance define the window where the algorithm should find the new peak. At the end of the procedure, a peak list (accurate mass - t_R *vs.* intensity) was generated and exported as .csv file. All the parameters used for the aforementioned processes in MZmine, are summarized in Table 4.

The serum data set from (-) ESI acquisition was treated in the same manner described above, using different parameter values, specific and related to the data, and a peak list (accurate mass - t_R *vs.* intensity) was again generated and exported as .csv file. All the MZmine parameters used in the serum data set from ESI (-) acquisition e.g. baseline correction; mass detection, chromatogram deconvolution, chromatogram builder, deisotoping, normalization and alignment are tabulated in Table S3-3.

Similarly, the urine data sets from (+) and (-) ESI acquisitions were processed through the MZmine toolbox, concluding to the generation of the corresponding peak lists (accurate mass - t_R *vs.* intensity). The parameters used are summarized in Table 4.

This method resulted in 3155 aligned features in serum (+) ESI and 2547 in serum (-) ESI data sets, while 2076 aligned features were detected in urine (+) ESI and 3046 in urine (-) ESI data sets.

Table SC3. MZmine parameters for the data preprocessing of serum and urine datasets from both (+) and (-) ESI-UHPLC-HRMS analyses.

	Serum		Urine	
	**(+) ESI**	**(-) ESI**	**(+) ESI**	**(-) ESI**
**Baseline correction**				
Type	base peak chromatogram	base peak chromatogram	base peak chromatogram	base peak chromatogram
Smoothing	500	500	500	500
Asymmetry	0.001	0.001	0.001	0.001
m/z bin width	1	1	1	1
**Mass detection**				
Algorithm	centroid	centroid	centroid	centroid
Noise level (cps)	1E4	3E4	8E4	1E5
**Chromatogram builder**				
Minimum time spam (min)	0.05	0.05	0.05	0.05
Minimum height (cps)	1E4	3E4	8E4	1E5
m/z tolerance (ppm)	5	5	5	5
**Chromatogram deconvolution**				
Algorithm	Wavelets (XCMS)	Wavelets (XCMS)	Wavelets (XCMS)	Wavelets (XCMS)
S/N threshold	5	5	5	5
Wavelet scales (min)	0.1-5	0.1-6	0.1-1	0.1-4
Peak duration range (min)	0.1-1	0.1-1	0.1-1	0.1-1
**Deisotoping**				
Algorithm	Isotopic peaks grouper	Isotopic peaks grouper	Isotopic peaks grouper	Isotopic peaks grouper
m/z tolerance (ppm)	5	5	5	5
t_R tolerance (min)	0.05	0.05	0.05	0.05
**Identification of adducts**	√	√	√	√
**Identification of peak complexes**	√	√	√	√
**Alignment**				
Algorithm	RANSAC	RANSAC	RANSAC	RANSAC
m/z tolerance (ppm)	5	5	5	5
t_R tolerance (min)	0.03	0.03	0.03	0.03
t_R tolerance (min) after correction	0.05	0.05	0.05	0.05
RANSAC iterations	15000	15000	15000	15000

Minimum number of points (%)	20	20	20	20
Threshold	4	4	4	4
Model	non linear model	non linear model	non linear model	non linear model
**Gap filling**				
Algorithm	Peak finder	Peak finder	Peak finder	Peak finder
Intensity tolerance (%)	80	80	80	80
m/z tolerance (ppm)	5	5	5	5
t_R tolerance (min)	0.05	0.05	0.05	0.05

The same MZmine procedure has been also performed using the "baseline cut-off" algorithm for the data deconvolution. The resulted peak lists were compared via the xMSanalyzer package in R (see section).

Microsoft Excel process.
The generated peak lists (accurate mass - t_R *vs.* intensity) have been exported to Microsoft Excel 2012 and manipulated using the CONCATANATE, ROUND and TRANSPOSE commands. The generated .xls file has been converted to .csv file or .txt files and was saved for further preprocessing.

xMSanalyzer.
The generated peak lists obtained from the different algorithms in the previous step, were imported to xMSanalyzer to compare the extracted features in terms of quality and similarity. Initially, following the appropriate commands, the two peak lists were compared via a Heatmap and the correlation of the common features were revealed. In a next step, a Venn diagram was used in order to determine the number of the unique and common features from the two selected algorithms and the aforementioned information was extracted in a .csv file for further process. Threshold values of 1 ppm for the *m/z* and 1 sec for the t_R, have been employed.

MetMSLine process.
The MetMSLine R script PreProc.QC.RLSC.GUI.R haw been employed. The *Number of Column Conditioning QC Samples at the beginning of acquisition* has been set to *5* and the *Quality control injection interval* has been set to 10. The *smoother span for LOWQESS signal attenuation smoothing* has been set to *0.2*, whereas features with standard deviation greater than *30%* employing the *Relative standard deviation cut-off for pooled QC signal filtration,* whereas the *alpha value for the generalized log transformation* is set to *1*.

MetaboAnalyst process.
The generated peak list (accurate mass - t_R *vs.* intensity) from the previous step was imported as .csv file, formatted according to the instructions[52], to the Metaboanalyst online metabolomics platform, to estimate and impute possible missing values in the sample peak list. Initially, rejection of features containing>20% zeros in both groups was performed. Then, the probabilistic principal component analysis (PPCA) was chosen in order to impute the possible missing values and applied to the initial .csv file. Therefore, a new peak list was generated, in which the missing values had been replaced by non-zero values. This new generated peak list (*.csv file) was used for the data analysis process.

In a next step, intensity normalization was performed to the peak list in order to adjust the differences among the samples. The normalization was achieved by choosing the option "normalization by a reference sample" and more specifically by averaging all the samples (pseudo-reference sample) in the control group. Following this approach, a normalized peak list (*.csv file) was generated, wherein the intensities of the different sample features were normalized. This normalized peak list was further

used for the data analysis process. The missing value estimation and the normalization process were also applied to the generated peak lists from all the data sets.

This method reduced the number of features to 1248 aligned features in serum (+) ESI and 1296 in serum (-) ESI data set, while 644 aligned features were detected in urine (+) ESI and 1228 in urine (-) ESI data set.

In a next step, after the missing value estimation process, the data were normalized applying the raw-wise normalization method, in order to reduce any systematic bias during the sample collection. More specifically, normalization was performed to the generated peak list (accurate mass - t_R *vs.* intensity) resulted from the different data sets, both serum and urine, in both (+) and (-) ESI modes, by a control sample each time, also known as the probabilistic quotient normalization. This method is considered as robust enough, to account for different dilution effects during sample preparation.

Metabolic pathway analysis.
Metabolic pathway analysis (MPA) was performed with MetPA (http://metpa.metabolomics. ca./MetPA/faces/Home.jsp) accessing the (a) Human Metabolome Data Base (HMDB) (http://www.hmdb.ca/), (b) METLIN Metabolomics Database (http://metlin.scripps.edu/index.php), (c) Kyoto Encyclopedia of Genes and Genomes (KEGG) (http://www.genome.jp/kegg/), (d) ChemSpider free chemical structure database (http://www.chemspider.com), (e) MassBank database (http://www.massbank.jp) and (f) LIPID MAPS (http://www.lipidmaps.org), to identify the affected metabolic pathways analysis and facilitate the visualization of the results. The significant identified features were imported as compound names or HMDB/KEGG ID's and possible associated biological pathways were investigated. Three parameters needed to be specified for pathway analysis: the specific pathway library, the algorithm for pathway enrichment analysis described above, and the algorithm for topological analysis. From the list of pathway libraries, *Homo sapiens* (human), which contains 80 pathways, was selected for MPA. The p-value (from pathway enrichment analysis) indicates the statistical significance of association of the altered metabolites with the pathway and the pathway impact value (from pathway topological analysis) is calculated as the sum of the importance measures of the matched metabolites normalized by the sum of the importance measures of all metabolites in the pathway. Those pathways with $p<0.05$ or an impact value>0.1 were considered and filtered out as potential target pathways.

Identification and structural elucidation of potential target metabolites.
The important features, which correspond to accurate mass - t_R, selected using the MVA approaches were putatively assigned to specific metabolites by match the selected features in terms of mass accuracy in online databases. More specifically, three publicly available databases of MS spectra of metabolites: (a) Human Metabolome Data Base (HMDB) (http://www.hmdb.ca/), (b) METLIN Metabolomics Database (http://metlin.scripps.edu/index.php), (c) Kyoto Encyclopedia of Genes and Genomes (KEGG) (http://www.genome.jp/kegg/), (d) ChemSpider free chemical structure database (http://www.chemspider.com), (e) MassBank database (http://www.massbank.jp) and (f) LIPID MAPS (http://www.lipidmaps.org) lipidomics gateway were used and furthermore the data were compared with those from previously published literature. Furthermore, additional data were used to confirm the proposed structures, such as the isotopic pattern and the RDB information. The matching criterion for the comparison of the expected isotopic pattern with the measured one was kept to 5% of matching, whereas the RDB, which provides the degree of molecule's unsaturation, was considered. Additionally, a deconvolution step has been performed to the corresponding spectra, using the appropriate software i.e., the Mass Frontier 5.0.1 and its freely available counterpart AMDIS (http://chemdata.nist.gov/dokuwiki/doku.php?id=chemdata:amdis), in order to gain information about a possible fragmentation that takes place in the source. All the putatively identified metabolites were further estimated through a t-test involving the abundance of the signal in the O and P groups,

with the aim to reveal any statistically significant differences concerning their means. The significant level has been set to 95%.

C. Statistical Data analysis

Principal component analysis.
PCA, being an unsupervised method, has been initially used to investigate the clustering of the QC samples in the corresponding analytical runs and to explain the percentage of variation. In addition, PCA has been also used to explore any trends or outliers in the data and to obtain an overview of variation among the groups. PCA was performed using both unit variance (UV) and Pareto scaling; confidence level on parameters was set at the 95% level whereas 200 maximum iterations have been employed. The scores values have been used for the evaluation of the results. The combination of R2 and Q2 values has been used in order to evaluate the optimal number of contributing parameters, as well as the scree plot. A cut-off value of 10% explained variance has been used for the optimal number of pc's kept for the construction of the PCA model.

Sparse principal component analysis.
sPCA, a variant of PCA, was performed initially for estimating a limited number of the most influential loadings (sparse loadings), allowing the efficient variable selection. sPCA was based on singular value decomposition (SVD) and sparsity is achieved via LASSO53,54 penalization. The value "TRUE" was set in the scale parameter, in order to obtain orthogonal sparse loading vectors, since in sPCA methods the orthogonality between the pcs and the loading vectors is lost. 100 variables were selected on each PCA dimension using the command KeepX. Cross-validation was performed to compute the R2 and Q2 values. The appropriate commands used in the mixOmics package implemented to the freely available R statistical language 3.0.2 for the competition of the sPCA analyses are tabulated in Supplementary method 2.

P**artial least squares–discriminant analysis**.
PLS-DA has been used in a supervised manner for further exploration of the difference between biomarker clusters and to display the maximum covariance of metabolic data with a defined Y variable (class) in the data set. The cross-validated cumulative Q2 (Q2cum) value was used as a degree of the predictive value of the PLS-DA model. A Q2 value of 1 indicates maximum predictive power, whereas Q2 values close to or below 0 indicate a lack of predictive power. As a rule of thumb, models with Q2cum>50% are of good predictive power in this context.

Orthogonal partial least squares–discriminant analysis.
OPLS-DA, a supervised pattern recognition approach, was used as a predictive model to discriminate the groups (classes) and to identify the differential metabolites in the different groups. The OPLS-DA model maximizes the covariance between the measured data of X variable (peak intensities) and the discriminant response of Y variable (class) within the groups. The quality of the model was determined by the goodness of fit and variation in the X (R2X) and Y (R2Y) variables and the predictability based on the fraction correctly predicted in a 1/7 cross-validation (Q2). Seven-round cross-validation was performed to eliminate the risk of overfit with only 1 predictive component for 2 classes. OPLS-DA shows more subtle changes in the occurrence and concentration of specific metabolites by focusing on compounds responsible for the discrimination between 2 groups.

Sparse partial least squares–discriminant analysis.
sPLS-DA was used as a sparse variant of the PLS-DA method, to allow for the selection of the most influential variables in accordance with the PLS-DA. Thus, 3 components (dimensions) were chosen, and the number of selected variables was set to 100. Variable selection was achieved using the LASSO

penalization on the pair of loading vectors. Cross-validation was performed to compute the R2 and Q2 values. sPLS-DA is based on the same concept as sPLS to allow variable selection, except that the variables are only selected in the X data set and in a supervised framework using non continuous variables, i.e., the X-variables with respect to different categories of the samples.
